# Effects of a School-Based Instrumental Music Program on Verbal and Visual Memory in Primary School Children: A Longitudinal Study

**DOI:** 10.3389/fpsyg.2012.00572

**Published:** 2012-12-21

**Authors:** Ingo Roden, Gunter Kreutz, Stephan Bongard

**Affiliations:** ^1^Department of Music, Carl von Ossietzky UniversityOldenburg, Germany; ^2^Department of Psychology, Goethe UniversityFrankfurt am Main, Germany

**Keywords:** music lessons, music training, verbal memory, visual memory, cognitive development, children, longitudinal study

## Abstract

This study examined the effects of a school-based instrumental training program on the development of verbal and visual memory skills in primary school children. Participants either took part in a music program with weekly 45 min sessions of instrumental lessons in small groups at school, or they received extended natural science training. A third group of children did not receive additional training. Each child completed verbal and visual memory tests three times over a period of 18 months. Significant Group by Time interactions were found in the measures of verbal memory. Children in the music group showed greater improvements than children in the control groups after controlling for children’s socio-economic background, age, and IQ. No differences between groups were found in the visual memory tests. These findings are consistent with and extend previous research by suggesting that children receiving music training may benefit from improvements in their verbal memory skills.

## Introduction

Previous research has examined whether and how musical abilities might influence other cognitive functions in cross-sectional (e.g., Cheek and Smith, 1999; Butzlaff, 2000; Schellenberg, 2011) as well as in longitudinal studies (Gardiner et al., 1996; Hyde et al., 2009; Schellenberg, 2009; Rickard et al., 2010; Moreno et al., 2011b). In particular, researchers have debated the benefits of music training on verbal and visual memory (Chan et al., 1998; Costa-Giomi, 1999; Kilgour et al., 2000; Ho et al., 2003; Jakobson et al., 2003; Forgeard et al., 2008; Piro and Ortiz, 2009; Rickard et al., 2010; Moreno et al., 2011a). Some studies suggest that music training is beneficial for verbal memory, but less so for visual memory, while other studies have produced conflicting evidence (Gardiner et al., 1996; Costa-Giomi, 1999; Jakobson et al., 2003). The present study was designed to assess some of these issues further within an extended longitudinal framework. In particular, verbal and visual memory skills of three cohorts of primary school children, one of which received instrumental training, were assessed three times over one and a half school years.

Musicians and non-musicians differ with regard to specific aspects of their brain structures and functions (Schlaug et al., 1995, 2009; Münte et al., 2002; Gaser and Schlaug, 2003; Moreno et al., 2009). For example, music training has been presumed to affect the development of the planum temporale (PT), a structure within the secondary auditory cortex. Whereas the left PT primarily mediates verbal memory (e.g., Frisk and Milner, 1990), the right PT primarily serves visual memory functions (e.g., Saykin et al., 1992). Based on the assumption of an asymmetric enhancement of the PT in response to music training, Ho et al. (2003) compared 90 male children between the age of 6 and 15 years who were learning an instrument (for 1–5 years) with schoolmates who had not received music training. Groups were matched in terms of age, education level, socio-economic background, and intelligence. Children in the music group achieved significantly higher scores in verbal but not in visual memory tasks. The music group learned approximately 20% more words from a 16-word list as compared to the non-music group. Moreover, children in the music group showed better verbal retention abilities (10-min delayed, 30-min delayed) than children in the control group. No significant differences were observed in the visual memory tests scores, however. Duration of music training and verbal learning performances correlated positively in this study, even after controlling for age and education level.

In a follow-up longitudinal study 1 year later, Ho et al. (2003) compared those children from the same cohort, who had just begun or continued their music training (for 1 year) with those who discontinued music training (at least 9 months prior to data collection). Children in the beginner and advanced music training groups significantly increased their verbal learning and retention performances, whereas no such increase was observed in the group who discontinued their music training. Remarkably, the verbal memory performances in the discontinued group remained stable at least 9 months after terminating the training program. Taken together, these findings indicate long-term enhancing effects of music training on verbal memory performances. Moreover, they support the hypothesis that musical training improves verbal rather than visual memory functions.

Although in the investigations referred to so far, no effects on visual memory were found, other studies reported improved visual memory performance for individuals who received music training (Rauscher et al., 1995; Bilhartz et al., 1999; Costa-Giomi, 1999; Hetland, 2000; Brochard et al., 2004; Sluming et al., 2007; Stoesz et al., 2007; Jakobson et al., 2008).

One reason for these different findings in the literature could be differences in methodological details. For example, studies on visual memory differ in demographic variables such as the participant’s age as well as in the duration and level of music training (from children with 1–5 years of training, Ho et al., 2003, to highly trained adults, Brochard et al., 2004). There is also inconsistency with respect to visual performance tasks across investigations. Whereas reaction time measures were used in one study (Brochard et al., 2004), Brandler and Rammsayer (2003), Ho et al. (2003) as well as Chan et al. (1998) examined recall accuracy. Moreover, participants in the latter study could have benefited from prior expertise with regard to the processing and recognition of complex visual designs by using an ideographic language system for reading and writing. Therefore, the fact that Chinese participants in the studies of Ho et al. and Chan et al. are highly skilled in processing complex visual designs may have inhibited positive effects of formal music training on visual memory.

Whereas studies cited so far were mainly based on private instrumental music lessons, only a few investigators looked at school-based music programs (e.g., Gardiner et al., 1996; Rauscher and Zupan, 2000; Rickard et al., 2010). For example, Gardiner et al. (1996) showed that children with a lower score on literacy and mathematics tests at baseline achieved similar scores on reading tests after 1 year of visual arts and music training at school as compared to control groups. They even outperformed controls in terms of mathematic achievement. However, the impact of music education remains unclear as music and art training were confounded in this study. Moreover, a non-musical comparison group with a different intervention was missing, which leaves open the possibility of a Hawthorne Effect. In a longitudinal study, Rickard et al. (2010) investigated a total of 142 children from nine regional state primary schools using tests of verbal learning and immediate verbal recall. Out of the entire group, 82 children participated in an enhanced school-based music program; the remaining 66 children served as controls and received standard music education. Participants were tested three times within the first 2 years of the study. In the third year a subset of the control sample (*N* = 44) was tested again. Children received either juggling training (*n* = 20) or continued standard music classes (*n* = 24) for about 1 year. Despite the differences among the time-displaced juggling, the music training group and the control group in terms of age, sample sizes, and duration of the training program, results showed that verbal learning and immediate verbal recall scores significantly increased after 1 year of school-based music training, whereas no such increase was found in the control group and in the juggling group. Nevertheless, these advantages disappeared in the second year and no effect was measured for the delayed verbal memory test at each time point. Moreover, no effects were observed in the visual recall test. However, results show a significantly enhanced visual perceptual score in the second year of the study for the music training group. In sum, mounting evidence suggests transfer effects from music training to non-musical cognitive benefits like verbal memory abilities (Strait and Kraus, 2011).

The present study examined the effects of a school-based music program on verbal and visual memory of primary school children over a period of 18 months. We hypothesized that children who participated in a school-based music program would significantly improve their verbal memory abilities (*Verbal Learning, Verbal Delayed Recall*, and *Verbal Recognition* values) over time as compared to their peers who received training in an unrelated area (natural sciences) or received curriculum-based music lessons only. In light of the conflicting results in previous work, we expected no differential effects on visual memory tasks between treatment and control groups.

## Materials and Methods

### Schools and participants

The sizes of groups of participants needed was calculated with the software program G*Power (Faul et al., 2007). According to this program, a total sample size of 54 participants was sufficient to obtain a medium effect size of Cohen’s *f* = 0.25 (Cohen, 1988) as a result from a repeated measures analysis of variance [ANOVA; within-between interactions; α-level: 0.05, Power (1 − β): 0.95, correlations among repeated measurements: 0.50]. However, a total of 73 children (mean age = 7.73 years; SD = 0.84, 37 male, 36 female) participated in this study. They were recruited from seven primary schools located in different parts of Germany. The 25 children (11 male, 14 female) in the music group received instrumental instruction through a special program established in primary schools in Germany. Children in this group were randomly chosen from six different classes out of three primary schools. Natural science group children (14 male, 11 female) were recruited from six different classes out of two primary schools that emphasized natural science skills. Finally, a total of 23 children drawn from another four classes in two primary schools with no additional training, served as controls.

Participants provided demographic information and were evaluated in terms of IQ, socio-economic background, and musical background, to determine any systematic differences in these variables prior to the intervention at group level (see Table 1).

**Table 1:** **Means (and SD) and test statistics of measures of the Musical Training (MT), Natural Science Training (NST), and No Training (CG) Groups at baseline**.

Variable	MT (*n* = 25)		NST (*n* = 25)		CG (*n* = 23)		*df*	*F-*value	*p*	$\eta_{p}^{2}$
	M	SD	M	SD	M	SD	
Age	7.32^a^	0.56	7.68^a,b^	0.70	8.22^b^	1.0	72	8.35	<0.001	0.19
IQ	114.68^b^	12.85	101.96^a^	12.78	107.6^a,b^	12.86	72	6.17	0.003	0.15
Parental income	2.63	1.59	2.29	1.57	2.54	2.03	45	0.17	0.85	0.01
Father’s educational level	2.93	0.26	2.82	0.96	2.90	0.88	46	0.10	0.90	–
Mother’s educational level	2.71	0.73	2.33	0.91	2.67	0.78	46	1.10	0.34	–
Cultural practice	3.17	0.99	3.14	0.94	2.92	1.19	52	0.25	0.78	–

*Same superscript indicates no significant differences; parental income: 0 = >20.000 €; 1 = 20.000–29.999 €; 2 = 30.000–39.999 €; 3 = 40.000–49.999 €; 4 = 50.000–59.999 €; 5 = <60.000 €; parents education level: 1 = did not go to school, no graduation; 2 = secondary modern school qualification (ISCED level 2); 3 = matriculation standard (ISCED level 3, 4); 4 = university degree (ISCED level 5); cultural practice: 1 = less than 10 books at home; 2 = 11–25 books; 3 = 26–100 books; 4 = 101–200 books; 5 = <500 books*.

The study was approved by the respective institutional review boards of the universities of Frankfurt and Oldenburg, Germany, and met ethical requirements for recruiting participants. Additional written informed consent for participation in the current study was obtained from school administrations, parents, and children. All schools participating in the music program needed to belong to a specific catchment area previously marked by the program administering foundation^1^. Furthermore, teachers from primary schools and music colleges were trained to administer pre-defined program standards. The allocation of children to the intervention groups (music and natural science) was not random. However, the selection of participants in the music group from several different cohorts ensured a wide distribution of socio-economic background variables and a reduction of systematic influences at school and class levels. Children in the control group participated in a natural science training program with the objective of promotion mathematics and general studies in primary schools. Hence math and general studies lessons at school were extended by the implementation of new educational standards consistently evaluated by a research program of the Leibnitz Institute for Science and Mathematics Education (IPN) in Kiel, Germany.

### Interventions

The music group children received weekly lessons for 45 min on musical instruments of their choice (guitar, violin, cello, flute, trumpet, clarinet, and drums). Depending on available resources, lessons were organized in different group sizes with a maximum of five. Thus the specific features of the instructional setting were subject to variation between schools. Nevertheless, the music program was entirely delivered by professionally trained instrumental teachers from public music schools. Standard elements of this training included singing, rhythm (clapping and percussion), and pitch identification exercises from first grade on. The instrumental instruction started from the second and was continued to the end of the fourth grade. Individual practice time was collected from the children, but due to high percentages of missing values as well as inconsistencies in the children’s self-reports, we refrained from using this information in the analyses. Nevertheless, parental responses indicated a certain amount of practice at home of each of the participating children, although the intensity of practice may have varied with respect to the specific domain.

Children in the natural science group received enhanced education in mathematics and general studies not confined to extra lessons but related to the school curriculum as a whole. Children in the second control group did not obtain additional training at school nor private instrumental lessons. Children in the natural science and in the no training groups still received curriculum-based music lessons at school. Assessments of additional extra-curricular school activities showed no significant differences among the three groups. Some uncertainty remains with respect to musical experiences of the children prior to the study. According to information provided by parents of the music group children, 12 children had participated in early music education programs. No such information was obtained from parents of children in the other groups.

### Measures

The German adaptation of Cattell’s Culture Free Intelligence Test (Cattell, 1961) by Weiß (2006, CFT-20R) was administered at T1 to measure fluid intelligence using its four subtests: series, classifications, matrices, and typologies. According to Weiß (2006) these subtests correlate highly with the “g”-factor of intelligence (*r* = 0.78 to 0.83). Results were assessed by raw means for each of the four subtests and standardized IQ scores adapted for age (*M* = 100, SD = 15). Socio-economic background was determined by standardized variables related to parental education, income, and the number of books in their homes. The latter is considered as a strong indicator for cultural practice (Bos et al., 2005; see Table 1). This information was acquired through questionnaires and telephone interviews with the parents. Musical background was assessed particularly with respect to extra-curricular activities including instrumental training in the control group just to make sure that no participants in the control groups (natural science training and no training) received any instrumental music training during the period of the study.

#### Verbal memory

To assess verbal memory performance, participants completed the German adaptation of Rey’s Auditory Verbal Learning Test (RVLMT; Rey, 1941) by Helmstaedter et al. (2001, verbal memory tests; VLMT). The VLMT was applied to assess *Verbal Learning*, *Verbal Delayed Recall*, and *Verbal Recognition* abilities. For each of five trials, participants were asked to listen and recall a list of 15 different words directly after hearing (*Verbal Learning*), followed by a 25-min delayed recall without read out (Verbal *Delayed Recall*). Finally, participants were requested to identify those 15 previously presented words out of a list of 30 words (*Verbal Recognition*). Retest-reliabilities reported in the manual range from *r_tt_* = 0.81 *(Verbal Recognition)*, *r_tt_* = 0.82 *(Verbal Learning)* to *r_tt_* = 0.87 *(Verbal Delayed Recall)*.

#### Visual memory

The standardized and computerized *Corsi Block Test* and *Matrix Span Test* by Hasselhorn et al. (2012) were used to access visual scribe and visual cache memory (for an overview to visual scribe and cache memory, see Logie et al., 1990). Both tests only minimally, if at all, evoke auditory recoding processes. Retest-reliabilities for both subtests ranged from *r_tt_* = 0.66 for 5- to 8-year-old children to *r_tt_* = 0.67 for the 9- to 12-year-old children.

The starting sequence of each subtest depended on the age of the subject. Eight-year-old children received a longer starting sequence span than the 7-year-old children. Both subtests based on an adaptive procedure with increasing (for a correct reproduction task) and decreasing (for an incorrect reproduction task) span tasks. Within these subtests the procedure was repeated until the last of the 10 sequences was finished.

In the *Corsi Block Test* children were asked to memorize and reproduce the path of a “smiley face” that moves randomly through an array of nine squares depicted on the computer screen. If the reproduction was correct for two trials, the movement of the “smiley” was increased by one step (to a maximum of nine) and the path between the “smiley” movements from square to square would get longer than before. If the recall failed for two trails the movement was decreased by one (to a minimum of two).

The *Matrix Span Test* displayed a pattern on a small chessboard with 16 fields, which disappeared after 4 s. Afterward children were asked to tag the remembered pattern on the touch screen. If they failed for two trials, the number of fields in the pattern was decreased by one (up to a minimum of two fields). Otherwise it was increased by one until the maximum of nine fields was reached.

### Procedure

Participants were tested individually in their classrooms in the course of one and a half hours. Assistants who were blind to the type of training (music, natural science, or no training) ran the data collection. The VLMT and the visual memory tests (Corsi Block Test and Matrix Span Test) were included in a larger research procedure encompassing an IQ test as well as standardized and demographic questionnaires. The IQ test and the standardized questionnaires had been conducted a day prior to the verbal and visual memory tests. The order of the verbal and visual memory tests was as follows: first, the *Verbal Learning* abilities were assessed. During the required break of at least 25 min after the *Verbal Learning* subtest, both tests for the visual memory abilities were administered (first the *Corsi Block Test* followed by the *Matrix Span Test*). Finally, children received the *Verbal Delayed Recall* and the *Verbal Recognition* tests. The order of all tests was the same for each child at all three points of measurement in order to avoid novelty effects.

Baseline measurements were conducted at the beginning of the school year (T1; October, 2009); the second measurement was taken at the beginning of the following school year (T2; October, 2010). The final measurement was completed at the end of the same school year (T3; July, 2011).

## Results

Table 1 shows the means, standard deviations, and test statistics for age, education, CFT-IQ scores, and socio-economic status for groups at the beginning of the study. Mean age and CFT-IQs differed significantly between groups at the beginning of the study. Children in the musical group were younger [95% CI (7.09, 7.55)] than children in the control group [95% CI (7.79, 8.65)]. They also earned higher CFT-IQ scores [95% CI (109, 120)] than children in the natural science group [95% CI (96.69, 107)]. There was no significant difference for IQ between music and control group children as well as between natural science and control group children. All groups were similar at the beginning of the study in terms of fathers’ and mothers’ educational level, parental income, and cultural practice. Gender was well balanced across groups (χ^2^ = 0.75; *p* > 0.69). Varying sample sizes are subject to missing data.

Repeated measures ANOVA were performed for the dependent measures of the verbal memory (*Verbal Learning*, *Verbal Delayed Recall*, and *Verbal Recognition Tests*) and the visual memory tasks (*Corsi Block Test* and *Matrix Span Test*). The quasi-experimental design was a mixed model, with Group as the between-subjects factor and Time as the within-subjects factor. Because of significant differences observed in the basic demographic variables (see Table 1), age and IQ were included as covariates within this model. Preconditions for conducting ANCOVAs were tested (normality and Mauchly’s test of sphericity) and were met in all cases except the Mauchly’s test, where the sphericity was significant for the verbal learning test and the verbal recognition test. Therefore, in the respective analyses the Greenhouse–Geisser correction for *p*-values and degrees of freedom was adjusted to calculate *F*-values. Mean values and standard deviations for all verbal and visual memory subtests were reported in Table 2.

**Table 2:** **Means (and SD) of verbal and visual memory assessment data for music, natural science, and control groups at three time points (T1–T3)**.

Measures	Music (*n* = 25)			Natural sciences (*n* = 25)			Control group (*n* = 23)		
	T1	T2	T3	T1	T2	T3	T1	T2	T3
Verbal learning	42.16 (8.63)	50.20 (10.05)	60.40 (7.46)	39.76 (9.53)	43.84 (8.77)	47.12 (8.43)	44.09 (7.97)	49.17 (9.26)	54.78 (7.60)
Delayed recall	10.04 (2.64)	11.64 (1.98)	12.80 (1.53)	9.68 (2.04)	10.72 (2.25)	10.60 (2.0)	10.09 (2.75)	10.48 (1.85)	11.35 (2.06)
Verbal recognition	11.32 (3.19)	13.16 (1.82)	14.32 (0.85)	11.72 (2.70)	12.72 (2.13)	12.76 (1.81)	12.13 (2.93)	12.87 (1.33)	12.91 (1.35)
Corsi block test	3.90 (0.54)	3.86 (0.83)	4.50 (0.76)	4.25 (0.84)	4.19 (0.71)	4.51 (0.83)	4.23 (0.61)	4.53 (0.59)	4.81 (0.83)
Matrix span test	4.21 (0.83)	4.63 (1.12)	4.95 (1.0)	4.26 (1.20)	4.76 (1.11)	4.69 (1.42)	4.66 (1.10)	5.23 (1.28)	6.01 (1.27)

**Figure 1:**
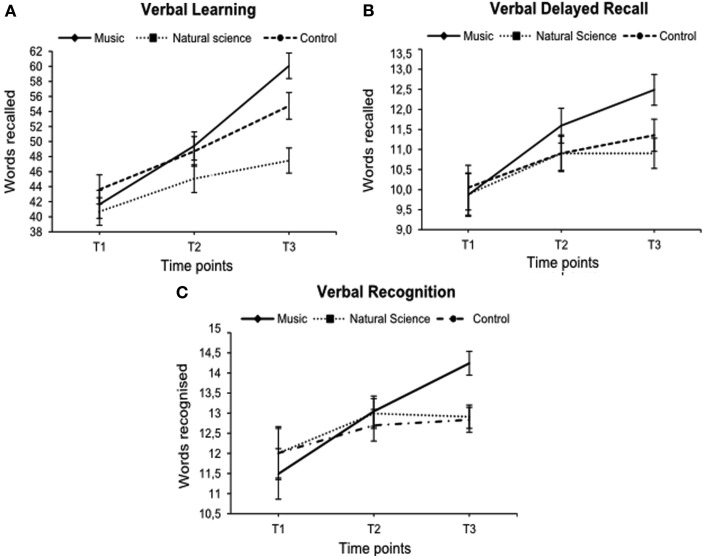
**Mean performance of the Verbal Learning (A), the Verbal Delayed Recall (B), and the Verbal Recognition Test (C) for experimental and control groups at baseline (T1), after 1 year (T2) and after one and a half years (T3) of music, natural science, or non-additional training (*N* = 73)**. Error flags indicate Standard Errors of Means (SEM).

Figure 1 reveals that across one and a half years, children in the music group showed a greater increase on every measure of the verbal memory than the natural science and the control group. Repeated measures ANCOVA revealed significant Time by Group effects for *Verbal Learning* [*F*(3.63, 123.4) = 5.19, *p* = 0.001, $\eta_{p}^{2}=0.13$], *Verbal Delayed Recall* [*F*(4, 136) = 3.17, *p* = 0.016, $\eta_{p}^{2}=0.09$] and for *Verbal Recognition* [*F*(3.05, 103.56) = 2.72, *p* = 0.048, $\eta_{p}^{2}=0.07$] test scores. However, no main effects on time were found for any verbal memory measures (all *F*s ≤ 1.95, *p* ≥ 0.15).

*Post hoc* analyses with pairwise comparisons at each time point of measurement showed that whereas groups did not differ at T1 [*F*(2, 72) = 1.48, *p* = 0.24, n.s.] the music group [95% CI (46.64, 53.77)] scored higher than the natural science group [95% CI (40.28, 47.41)] in the *Verbal Learning Test* at T2 [*F*(2, 72) = 3.62, *p* = 0.032, $\eta_{p}^{2}=0.09$]. Furthermore, the music group [95% CI (57.27, 63.53)] outperformed natural science group [95% CI (43.99, 50.25)] and control group [95% CI (51.52, 58.04)] at T3 [*F*(2, 72) = 18.03, *p* < 0.001, $\eta_{p}^{2}=0.34$]. Similarly, the music group [95% CI (12.05, 13.55)] showed superior performance in *Verbal Delayed Recall* at T3 [*F*(2, 72) = 8.91, *p* < 0.001, $\eta_{p}^{2}=0.20$] compared to the natural science group [95% CI (9.85, 11.35)] and the control group [95% CI (10.57, 12.13)]. Finally, the music group [95% CI (13.76, 14.88)] scored higher than the natural science group [95% CI (12.20, 13.32)] and the control group [95% CI (12.33, 13.49)] in the *Verbal Recognition Test* at T3 [*F*(2, 72) = 9.43, *p* < 0.001, $\eta_{p}^{2}=0.21$]. The observed effects in the music group were large: effect sizes of the improvement for all verbal memory measures at T3 were large (for an overview of effect size benchmarks, see Cohen, 1988).

Subsequent comparison of means for within subject effects with adjusted alpha level (*p* = 0.05/2 = 0.025) showed that the music group significantly improved their performances in the *Verbal Learning* [*t*(24) = 5.71, *p* < 0.001, *d* = 1.16], *Verbal Delayed Recall* [*t*(24) = 5.66, *p* < 0.001, *d* = 1.27], and *Verbal Recognition* [*t*(24) = 3.09, *p* = 0.005, *d* = 0.67] test scores from T1 to T2, and from T2 to T3 [*Verbal Learning* *t*(24) = 6.43, *p* < 0.001, *d* = 1.35; *Verbal Delayed Recall* *t*(24) = 4.92, *p* < 0.001, *d* = 1.06, and *Verbal Recognition* *t*(24) = 3.01, *p* = 0.006, *d* = 0.65]. In contrast, participants in the natural science group only improved their verbal memory performances in the *Verbal Delayed Recall* score [*t*(24) = 2.46, *p* = 0.021, *d* = 0.49] from T1 to T2. The control group showed significant increases for the *Verbal Learning* Score from T1 to T2 [*t*(22) = 5.14, *p* < 0.001, *d* = 1.07] and from T2 to T3 [*t*(22) = 4.22, *p* < 0.001, *d* = 0.88] notwithstanding that the increases were smaller than in the music group. Finally, the control group significantly improved their performances in the *Verbal Delayed Recall* score only from T2 to T3 [*t*(22) = 3.33, *p* < 0.05, *d* = 0.70].

**Figure 2:**
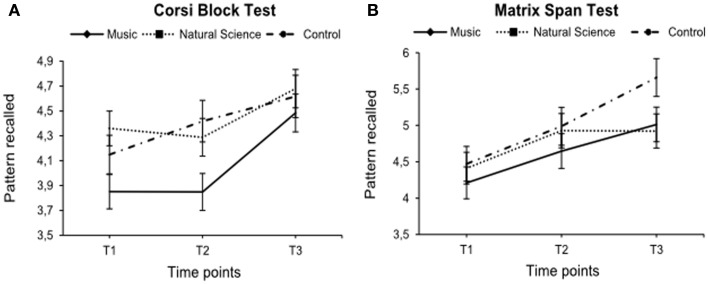
**Mean scores of the Corsi Block Test (A) and the Matrix Span Test (B) for the experimental and control groups (*N* = 73) at baseline (T1), after 1 year (T2) and after one and a half years (T3) of music, natural science, or non-additional training**. Error flags indicate Standard Errors of Means (SEM).

For the visual memory tests, no interaction or main effects were found across the study (Corsi Block Test: all *F*s ≤ 2.74; *p* ≥ 0.07; Matrix Span Test: all *F*s ≤ 1.26; *p* ≥ 0.29). The respective values for these memory tests are displayed in Figure 2.

## Discussion

This study was designed to investigate the effects of a school-based music training program on the development of verbal and visual memory skills in primary school children. We hypothesized that over a period of one and a half years, musically trained children would significantly outperform both a comparison group of children obtaining enhanced natural science training and a control group without specific training, with respect to measures of verbal memory performance. Furthermore, we did not expect to find any differences between groups in their performances concerning visual memory abilities. Both of these hypotheses were confirmed according to the present results.

First of all, although performance levels in memory tests were similar at baseline, children receiving music instruction scored significantly higher in tests addressing verbal memory as compared to controls, after 18 months of instrumental training. Specifically, music-trained children showed significant increases in the *Verbal Learning Test* score over the whole testing period, whereas no such improvement was observed in the natural science and the non-training groups. These patterns of changes prevailed after adjustment of the model to account for influences of individual IQ and age. In addition, the music training group scored significantly higher in the *Verbal Delayed Recall Test* and in the *Verbal Recognition* *Test* after 18 months and thus outperformed the natural science and the non-training group. These results suggest that musically trained children developed more efficient short-term (verbal learning skills) and long-term (verbal delayed recall and verbal recognition skills) memory strategies as compared to their peers in the two control groups. The large effect sizes observed in the music group for all verbal memory measures at the last time point (T3) strengthen our interpretation. Moreover, musically trained children continuously improved their performances over time (from T1 to T2 and from T2 to T3) in each of the three verbal memory measures, whereas such an increase was found neither in the natural science nor in the non-training group.

The findings obtained here are consistent with previous quasi-experimental research (Chan et al., 1998; Kilgour et al., 2000; Ho et al., 2003; Jakobson et al., 2008) which showed that musically trained children significantly improved their verbal memory abilities as compared to control groups. The current study extends these findings by showing the same effect on verbal memory performances during a school-based music program within a longitudinal design. It is noteworthy that effect sizes for the three verbal memory tests in the present study were higher than expected from prior research (e.g., Ho et al., 2003; Rickard et al., 2010). Hence the large effect sizes strengthen our confidence in the relationship between instrumental music training and verbal memory abilities examined in our study.

### Differences in culture, research methods, and musical experiences

Jakobson et al. (2008) suggested that the lack of effects in visual memory performance as observed by Chan et al. (1998) and Ho et al. (2003) could be explained by cultural differences. Both investigators worked with native Asian participants, who are trained in an ideographic reading and writing system. This training was found to be associated with improved memory for abstract designs but not for words (e.g., Flaherty, 2000). Therefore it was suggested that formal music training may have provided no additional benefit for visual memory performances in these groups. However, such cultural influences appear unlikely with respect to the present findings, because our participants were trained in an alphabetical rather than ideographic language system. The present results are in line with Rickard et al. (2010), who found no significant improvement in simple visual memory abilities (visual recall) for music-trained children in another Western (Australian) sample of participants.

Without denying possible cultural influences on certain domains of cognitive performance, we suggest that the discrepancy in the results reported for the relationship between formal music training and verbal memory on the one hand, and between formal music training and visual memory on the other, lie in individual music training histories as well as in the different performance measures for visual memory abilities. For example, Brandler and Rammsayer (2003), Chan et al. (1998), Jakobson et al. (2008), and Kilgour et al. (2000) examined highly trained adults (from 10 to 14 years of formal music training) whereas Ho et al. (2003), Brochard et al. (2004), and Rickard et al. (2010) investigated children with relatively restricted music training experiences (to a maximum of 5 years). Moreover, visual memory in prior research had been assessed by a variety of measures including reaction time (Brochard et al., 2004), accuracy of recall (Chan et al., 1998; Ho et al., 2003; Rickard et al., 2010), topographical memory skills (Brandler and Rammsayer, 2003), visual perception ability (Brochard et al., 2004; Rickard et al., 2010), and visual-auditory memory (Moreno et al., 2011b).

### Private music lessons vs. school-based music training

Another factor, which could explain diverging findings between the current results and previous investigations for visual memory abilities of musically trained individuals (e.g., Jakobson et al., 2008), might be the nature of a school-based music training program, and the associated instructional setting. Typically, group-based training appears to place stronger emphasis on listening skills, because all members of the group need to monitor and respond to acoustic information from different sources (teacher, fellow students). By contrast, fewer demands should arise with respect to score reading, which must accommodate the skills of the weaker score readers. Therefore, specific advantages for visual cognition might only occur in more intensive private music lessons (see Rickard et al., 2010).

Importantly, the implementation of an alternative school-based natural science training program as a comparison group should have accounted for novelty effects in the target group (controlling for paying more attention and additional training to the treatment group). Hence, a school-based music training program suggests similar effects on verbal memory abilities as has been shown for private instrumental instruction in previous work.

### Neurocognitive mechanisms of verbal memory effects

The most likely interpretation of our findings is that musically trained children develop particularly efficient memory strategies for materials (here: words), which are represented as auditory codes. Playing music requires continued monitoring of meaningful chunks of information. Rather than individual notes, these chunks entail clusters of notes that are combined into meaningful melodic gestures and phrases under the influence of Gestalt principles as well as syntactic rules that are common to Western tonal music (Lerdahl and Jackendoff, 1983). Therefore, the basic units on which cognitive processes operate in the case of musical materials are “word-like” clusters of notes, which comprise melodies that may extend well beyond the limits of working memory. Although musical motifs do not entail denotative meaning as do words (Koelsch et al., 2004), they do have a quasi-syllabic structure, and, notably, they are based on a temporal framework that entails metric structures (weak and strong beats in music are paralleled by stresses on syllables in language; see Patel and Daniele, 2003). Such timing properties are thought to be important in the representation of materials stored in working memory (Jakobson et al., 2003). Neuropsychological studies suggest a correlation between variables related to the years of music training, the age of onset of music training and the amount of practice on the one hand, and the degree of structural or functional enhancement in specific brain areas, on the other (Pantev et al., 2001; Bengtsson et al., 2005). For example, the studies by Chan et al. (1998) were based on the hypothesis that music training affects the neuroplasticity in auditory regions of the temporal cortex, especially in the left PT (Schlaug et al., 1995), which is still consistent with enhanced auditory temporal processing ability (Jakobson et al., 2003). Williamson et al. (2010) maintained that short-term memory for tones has several similarities to verbal memory. The authors suggest that verbal and musical sounds are processed in the auditory short-term memory. In the papers by Kraus and Chandrasekaran (2010) and Strait and Kraus (2011) the authors argued that music is a powerful tool for modeling neural structures and functions via general auditory processing (including the processing of speech). Hence it might be possible that musicians would also gain an advantage in their verbal memory performances.

However, further research will be necessary to clarify the conditions under which music training might affect verbal and visual memory in music and non-music groups.

### Instrumental training and child literacy

Previous research showed that music training might promote cognitive mechanisms that underlie child literacy and language development (Besson et al., 2011; Moreno et al., 2011a; Strait and Kraus, 2011) as well as reading abilities (Anvari et al., 2002; Moreno et al., 2011b). Such mechanisms include auditory cognitive skills such as verbal or auditory working memory (Tierney et al., 2008; Kraus and Chandrasekaran, 2010; Williamson et al., 2010; Roden et al., in press). Supporting the interpretation of common processes in music and speech, Bidelman et al. (2009) investigated the influence of domain-specific experience of music and language on the encoding of music (musical intervals) and on speech (lexical tones) pitch processing. Brainstem frequency-following responses in native Chinese, English musicians, and English non-musicians were recorded while the participants listened to homologs of musical intervals and lexical tones. Results showed that pitch experience in music or language could be transferred from one domain to another. Compared to the group of English non-musicians, both Chinese and English musicians showed a higher pitch tracking accuracy and pitch strength. However, some interesting difference was found between the Chinese and English musicians: the Chinese group more focused on the rapid changes of pitch that were similar in Mandarin Chinese language, whereas English musicians were more sensitive to the parts of the stimuli similar to the notes of musical scale. Hence, the authors argue that pitch encoding in the auditory brainstem is domain-general for music and language but different in pitch extraction mechanisms. Further studies presented more evidence for a relationship between musical abilities and phonological processing (Anvari et al., 2002; Moreno et al., 2009; Dege and Schwarzer, 2011). Along those lines we found that music-trained children showed greater improvements in tasks addressing the phonological loop (word span test and non-word recall test) than non-music children over a period of one and a half year (Roden et al., in press).

In sum, prior results argue for a positive transfer effect from musical expertise onto speech and language processing. The three reported measures of the auditory verbal memory test used in the present study extend the above findings by showing that children receiving music lessons demonstrated larger improvements of verbal memory skills than the control group children, even after IQ was controlled. Our results provide additional converging support to recent findings due to the fact that children in the music group did not differ on average from the control groups on any verbal memory measure at the beginning of our study (T1), and that there was no significant difference observed in the visual memory tests at any time point (T1, T2, and T3). They further extend the previous finding of Tierney et al. (2008), showing that musical experience may affect short-term verbal memory abilities (immediate verbal rehearsal processes) as well as long-term verbal memory skills (delayed verbal recall and verbal recognition) in children.

### Limitations and conclusion

Considering the range from medium to high effect sizes of all interactions for the verbal memory tests used in the present study, these results suggest some validation of a transfer effect from music to more specific cognitive domains. Nevertheless this interpretation must be treated with caution. The quasi-experimental design of this study suggests that participant groups were self-selected rather than constituted by chance. Experimental studies based on completely randomized trails should provide more persuasive evidence for the benefits of music training. However, the inclusion of two comparison groups and three time points of measurements may have diluted some of the self-selection bias. Although the same tests were used for each child at all three points of measurement, it is unlikely that practice effects would account for our results. In particular, no main effects on time were found for any verbal or visual memory measures. Therefore, the interaction effects suggesting benefits for the music group in the verbal memory measures could be attributed to effects of the intervention rather than of practicing.

There are some further limitations to be considered in the present study. Firstly, the size of the group of children receiving music training was limited to five, whereas the natural science training involved all children in one school-class. However, at T2 and T3 the group size for music training increased by teaching the third and fourth graders together. Secondly, the individual practice time for children in the music group showed at home is uncertain due to high percentages of missing values as well as inconsistencies in the children’s self-reports. In addition, the available data concerning the participation in teaching and learning activities at home was insufficient to clarify any bias on our reported results across groups. Nevertheless, it is unlikely that such activities would only affect the verbal memory and not the visual memory skills. Similarly, it is possible that the general learning activity in the three groups differed systematically, thus providing a bias that has been undetected so far. Nevertheless, we observed no differences at baseline in the verbal and visual memory tests between groups, which indicate, that the bias of individual practice time or learning activities could not be responsible for the reported development in the verbal memory measures. Finally, to approximate exposure to instruction outside of the classroom, we checked the extra-curricular activities of each of the children in the three groups and we did not find any differences among the groups.

Taken together, school-based instrumental training was shown here to affect memory for verbal rather than visual materials. The study corroborates and extends notions of transfer effects of cognitive skills from the music to the linguistic domain. These findings are consistent with previous experimental and quasi-experimental work (Chan et al., 1998; Ho et al., 2003; Rickard et al., 2010). To our knowledge, the present data extend these findings in that they are the first to show that components of the verbal memory (verbal learning, verbal delayed recall and verbal recognition) in primary school children increases over a time period of 18 months in response to a school-based music training program. Although the mechanisms that drive these effects remain unclear, the findings reinforce the more general notions of beneficial effects of music education for children at primary schools, and, possibly, pre-schools in terms of their cognitive development, in general and language acquisition in particular.

## Conflict of Interest Statement

The authors declare that the research was conducted in the absence of any commercial or financial relationships that could be construed as a potential conflict of interest.

## Footnotes

^1^The musical training program is guided by a foundation called “Jedem Kind ein Instrument” (JeKi), [An Instrument for Every Child]. For further information, please visit www.jedemkind.de.
